# Overexpression of MCL-1 in canine hepatocellular carcinoma and its efficacy as a prognostic marker

**DOI:** 10.1186/s12917-025-04798-6

**Published:** 2025-05-16

**Authors:** Jehun Baek, Jaeho Cho, Hun-kyeong Shin, Wan Hee Kim

**Affiliations:** Department of Veterinary Clinical Sciences, College of Veterinary Medicine, Research Institute for Veterinary Science, Seoul National University, 1 Gwanak-Ro, Gwanak-Gu, Seoul, 08826 Republic of Korea

**Keywords:** Canine, Western blotting, Immunohistochemistry, Hepatocellular carcinoma, MCL-1

## Abstract

**Background:**

Myeloid cell leukemia-1 (MCL-1)—an anti-apoptotic protein of the B-cell lymphoma 2 family—is commonly overexpressed in human cancers, promoting tumorigenesis and chemoresistance. Upregulated MCL-1 in human hepatocellular carcinoma (HCC) has been demonstrated in numerous studies, and therapeutic agents targeting this protein have been assessed. However, its prognostic significance in canine HCC remains unclear. The objective of this study was to detect MCL-1 protein in canine normal liver tissue and compare its expression level with that in HCC tissue using western blotting. Immunohistochemistry (IHC) was used to quantify MCL-1 intensity levels in normal, non-neoplastic hepatic diseases, and HCC tissues, and the differences were assessed. Additionally, the relevance of MCL-1 immunostaining to various clinical and pathological parameters was evaluated.

**Results:**

MCL-1 expression was markedly elevated in HCC tissues relative to normal liver tissues (*P* = 0.029). Additionally, all 10 normal liver tissues exhibited low IHC expression, which significantly increased as the malignancy progressed (*P* < 0.001). In the HCC samples, high MCL-1 immunostaining was substantially correlated with metastatic status (*P* = 0.034) and tumor size (*P* = 0.046). Moreover, survival curve analysis revealed a significant relationship between upregulated MCL-1 and lower disease-free survival and overall survival rate (*P* = 0.006 and *P* = 0.031, respectively).

**Conclusion:**

MCL-1 expression is increased in canine HCC, and its overexpression significantly correlates to worse clinical outcomes. Therefore, MCL-1 is considered to be a promising prognostic marker.

**Supplementary Information:**

The online version contains supplementary material available at 10.1186/s12917-025-04798-6.

## Background

Primary hepatic tumors are uncommon in dogs and account for < 1.5% of reported malignancies. The most prevalent hepatic neoplasm is hepatocellular carcinoma (HCC), accounting for up to 77% of all canine primary hepatobiliary neoplasms [1]. HCC can be classified into three distinct subtypes: massive, diffuse, and nodular. Prognosis is contingent on the specific morphological subtype [2]. The massive subtype typically has low metastatic potential and is often amenable to surgical resection with a favorable prognosis [3]. Conversely, the diffuse and nodular subtypes are associated with a significantly high incidence of metastasis, with rates reaching up to 93%. These subtypes often involve multiple hepatic lobes, significantly limiting surgical resection as a treatment option [2–4]. Systemic chemotherapy, such as gemcitabine and carboplatin, was administered to dogs with unresectable HCC; however, these applications failed to demonstrate significant survival benefits [5]. Sorafenib has exhibited significant anti-tumor effects in dogs with advanced HCC [6]. However, the efficacy of conventional systemic chemotherapy and other treatments, such as trans-arterial embolization and radiotherapy, remains inadequately studied in the veterinary literature, highlighting the need for further prospective research.

The apoptotic pathway, which is regulated by intrinsic and extrinsic pathways, maintains cellular homeostasis, guides tissue development, and prevents tumorigenesis. The BCL-2 family mainly controls the intrinsic pathway [7]. This family is categorized into anti-apoptotic, Bcl-2 homology (BH) 3-only, and pro-apoptotic proteins according to their structural and functional properties [8].

Myeloid cell leukemia-1 (MCL-1) is an anti-apoptotic protein of the Bcl-2 family [7, 8]. The intrinsic apoptosis mechanism involves the upregulation of BH3-only proteins that neutralize the anti-apoptotic proteins, including MCL-1. This cascade activates pro-apoptotic protein and stimulates the release of cytochrome c from mitochondria by increasing mitochondrial outer membrane permeability (MOMP) [9]. The released cytochrome c promotes oligomerization of APAF-1 that recruits pro-caspase-9 to create apoptosomes. This formation triggers the autocleavage and activation of caspase-9, resulting in apoptosis [9, 10]. The MCL-1 protein is essential for cell survival and serves a pivotal function in liver tissues by maintaining hepatocellular homeostasis and protecting hepatocytes from apoptotic processes [11].

Apoptosis resistance is a characteristic of cancer pathophysiology [12]. MCL-1 has significant oncogenic potential and is frequently upregulated in various human cancers, including solid tumors and hematologic malignancies [8, 13–16]. Additionally, MCL-1 overexpression has been correlated with local recurrence and shorter overall survival across multiple cancer patients [13–16]. MCL-1 is upregulated in human HCC tissues, representing that MCL-1 is a crucial survival parameter in HCC [17–19]. These findings imply that MCL-1 might be an effective prognostic indicator and treatment target [10], and clinical trials are underway investigating the effectiveness of MCL-1 inhibition in cancer treatment [20]. In veterinary studies, enhanced MCL-1 expression was described in canine mast cell tumor cell lines [21], and its prognostic significance has been reported in mammary gland tumor (MGT) tissue [22]. However, the role of MCL-1 in canine HCC has not been identified.

This study assessed the presence of MCL-1 protein in canine liver tissue using western blotting. Additionally, we evaluated MCL-1 expression levels in the normal, non-neoplastic hepatic disease, and HCC tissues using immunohistochemical analysis. Moreover, we assessed the association between MCL-1 immunostaining and clinicopathological parameters to explore the possible utility of MCL-1 as a prognostic marker. Overall, our results may serve as a foundation for future research on developing targeted therapeutics for canine HCC.

## Methods

### Tissue sampling

Liver tissues were collected from five healthy beagles residing at the Department of Veterinary Medicine, Seoul National University as a control group. The health of control animals was confirmed to be normal through physical examination, blood tests, radiography, and abdominal ultrasonography before euthanasia. To minimize pain, meloxicam (0.2 mg/kg) was administered intravenously (IV). All dogs were euthanized via 10% potassium chloride, which was administered under propofol induction and maintained under general anesthesia with isoflurane. These dogs were euthanized for reasons independent of the current study (SNU-200709-4-5); therefore, this did not affect our results. Two liver samples were aseptically collected from different liver lobes of each dog using the guillotine technique. Overall 10 normal liver tissues were fixed with 10% neutral-buffered formalin and embedded in paraffin blocks. Additionally, four normal samples were immersed in a liquid nitrogen storage box and stored at -80 °C immediately. These tissues were confirmed to be normal through histopathological examination using hematoxylin and eosin staining.

Between October 2018 and July 2024, non-neoplastic hepatic (*n* = 30) and HCC (*n* = 30) samples were obtained from 60 client-owned patients who underwent surgical procedures at the Veterinary Medical Teaching Hospital of Seoul National University. Half an hour before surgery, patients were administered cefazolin IV (22 mg/kg). Premedication through constant rate infusion (CRI) of remifentanil-midazolam-ketamine (RMK) complex was then applied. Propofol was utilized to induce general anesthesia, and dogs were maintained by isoflurane. RMK CRI was administered during and after surgery for pain management. All HCC diagnoses were confirmed according to the results of histopathological examinations performed at IDEXX Laboratory or the Veterinary Pathology Laboratory at Seoul National University. A small segment of the excised tissues were sampled after surgery. All samples underwent fixation and embedding via the same procedure as normal tissues for immunohistochemical analysis. For subsequent western blot analysis, certain HCC tissues were frozen at -80 °C. Patient owners were aware of the study’s procedures and purposes, and written consent was provided before participation.

Clinical information was collected on patient signalment, blood analysis, tumor location, tumor size, morphological subtype, metastasis status, and local recurrence. The canine liver lobes were divided into three primary divisions. The left division consists of the left lateral and medial lobes. The right division is composed of the right lateral and caudate lobes, whereas the quadrate and the right medial lobes comprise the central division [23]. Hepatic tumors are classified into the following morphological subtypes. The massive type is defined by a large single mass localized within one liver lobe, occasionally metastasizing to other lobes. The nodular type is marked by multiple nodules in numerous liver lobes. The diffuse hepatic tumors may involve widespread neoplastic nodules throughout entire hepatic lobes or extensive infiltration of liver parenchyma [2]. All dogs underwent radiology and ultrasound or computed tomography. Dogs with other pre-existing malignancies or confirmed metastasis before surgery were excluded. The Seoul National University Institutional Animal Care and Use Committee ethically approved the study protocol and procedures (SNU-240311-2).

### Western blotting

For Western blot analysis, four paired samples of normal and HCC tissues with sufficient protein preservation were selected to reflect variation in tumor size and metastatic status. Proteins were homogenized and extracted with RIPA buffer (Thermo Fisher Scientific), which is combined with protease inhibitor (Sigma-Aldrich). Quantification of the proteins was performed by the BCA Protein Assay Kit (Bio-rad). Protein samples (30 µg) were combined with 4X Laemmli’s sodium dodecyl sulfate (SDS) sample buffer (GenDEPOT) and heated at 95 °C for 5 min for denaturation. SDS-polyacrylamide gel (10%; SMOBIO Technology) separated equal amounts of denatured proteins, which were subsequently transferred to a polyvinylidene difluoride membrane (GenDEPOT, 0.45 μm). After treating the membranes with 5% skim milk for 1 h, they were reacted with the primary antibody overnight at 4 °C. Rabbit polyclonal anti-MCL-1 antibody (1:1500 dilution, Abcam, #ab28147) and mouse monoclonal anti-β-tubulin antibody (1:3000 dilution, Santa Cruz Biotechnology, #sc-166729) were used as the primary antibodies. Subsequently, the membranes were incubated with the following secondary antibodies—anti-rabbit IgG horseradish peroxidase (HRP)-linked antibody (1:3000 dilution, Cell Signaling Technology, #7074) for MCL-1 and anti-mouse IgG HRP-linked antibody (1:3000 dilution, Cell Signaling Technology, #7076) for β-tubulin—at room temperature for 1 h. The ECL reagent (Advansta) was employed for protein detection, and ImageQuant LAS 4000 mini (GE Healthcare Biosciences) was used to capture the images (see Additional file 1).

### Immunohistochemistry

A microtome (Leica Microtome HM355S) was used to section paraffin-embedded tissues into 3 μm slices. Sectioned tissues were incubated at 60 °C and deparaffinized twice with xylene for 5 min each. Tissue sections were rehydrated using an ethanol series (100%, 100%, 90%, 80%, and 70%) and phosphate-buffered saline (PBS), with each step lasting 5 min. Antigen retrieval was conducted using 10 mM citric acid buffer (pH 6.0) in 2100-antigen retriever (PickCell Laboratories) for 20 min. To prevent the activity of endogenous peroxidase, tissue sections were treated with hydrogen peroxide (3%) for 30 min. Normal goat serum (10%; Abcam, #ab7481) was used on the sections for 20 min. Subsequently, they were exposed to rabbit polyclonal anti-MCL-1 antibody (1:700 dilution, Abcam, #ab28147) in a moist environment overnight at 4 °C. Negative control tissue was treated with a rabbit IgG isotype control (1:700 dilution, Cell Signaling Technology, #3900) to exclude the non-specific activity of the secondary antibodies. As a positive control, we used a canine MGT sample [22]. Sections were treated with a biotinylated goat anti-rabbit IgG secondary antibody (Abcam, #ab64256) and incubated for 10 min at room temperature. This was further incubated with streptavidin-HRP (Abcam, #ab64269) for 10 min under similar conditions. They were then processed using a 3.3’-diaminobenzidine (DAB substrate kit, Abcam, #ab64238) for 5 min to enable visualization of the antigen. Using Mayer’s hematoxylin solution, the slides were counterstained. Dehydration through an ethanol series (90%, 100%, and 100%) and cleared with xylene twice. PBS washes were performed between each step of the procedure. An Olympus microscope (BX50F4) equipped with a Tucsen digital camera (FL-20) was used to examine and capture images of the stained slides.

### Quantification of immunostaining

MCL-1 expression was assessed semiquantitatively by multiplying the immunostaining proportion and intensity scores. These scores were assessed using the MCL-1 scoring criteria documented for canine MGT [22]. Staining intensity was classified as 0 (negative), 1 (weak), 2 (moderate), and 3 (strong). Stained cell proportion was quantified by evaluating a minimum of five individual high-power fields, analyzing over 1000 cells to assign the corresponding intensity level accurately. The final histoscore (maximum score: 300) was calculated using the following formula: (0 × % negatively stained cells) + (1 × % weakly stained cells) + (2 × % moderately stained cells) + (3 × % strongly stained cells). The receiver operating characteristic curve was employed to obtain the optimal cut-off score. The score for classifying MCL-1 expression as high or low was precisely determined to be 187, which maximized diagnostic accuracy with a sensitivity of 0.7 and specificity of 0.825. Two observers (Jehun Baek and Jaeho Cho) independently assessed the scores with the histoscores anonymized. The intraclass correlation coefficient was used to quantify interobserver agreement, which was > 0.9, indicating high consistency of assessment.

### Follow-up data

All patients returned for follow-up within 14 days of surgery and continued to be monitored every 3 to 6 months thereafter, depending on the circumstances. Physical examination, radiography, and abdominal ultrasound were conducted to check for metastasis and recurrence. Additional diagnostic tests, such as computed tomography, fine-needle aspiration, and biopsy were performed if necessary.

### Statistical analysis

The significant difference between the relative MCL-1 levels in normal and tumor tissues, measured based on western blot analysis, was evaluated using the Mann–Whitney *U* test. A linear-by-linear association test was performed to assess the association between MCL-1 immunostaining level and liver tissue types: normal, non-neoplastic hepatic disease, and HCC. The correlation between clinicopathological parameters and MCL-1 expression was assessed using Fisher’s exact and chi-square tests. Age normality was evaluated using the Shapiro–Wilk and Kolmogorov–Smirnov tests. As the results satisfied the normality, Student’s *t*-test compared variables according to the MCL-1 immunostaining result. Kaplan–Meier curve was generated and analyzed to assess disease-free survival (DFS) and overall survival (OS), with comparisons performed with the log-rank test. The definition of DFS was the duration between the first surgery and the onset of either metastasis or recurrence. Cases were right-censored if they were lost to follow-up or died, and if there was no evidence of metastasis or recurrence. The period from the first surgical resection to death resulting from HCC was described as OS. In the OS analysis, dogs were considered right-censored if they died from unrelated causes, were alive at the study’s endpoint, or did not participate in follow-up. Multivariate Cox proportional hazards regression analysis was performed to assess the prognostic value of MCL-1 expression while adjusting for potential confounding effects of other clinicopathological variables. Statistical analysis was performed using SPSS (v.29.0, IBM Corp.) and GraphPad Prism 8 software (GraphPad, Inc.). Significance was set at *P* < 0.05.

## Results

### Clinical and histological data

This study included 65 dogs. The signalment and histological type of each tissue are summarized in Table 1. According to the World Small Animal Veterinary Association, non-neoplastic hepatic disease tissues can be classified into three categories: inflammatory lesions, reversible hepatocellular injury (vacuolar hepatopathy), and other pathological conditions, as determined by histopathological assessment [24]. The HCC samples were further categorized into morphologic subtypes, showing that the massive subtype (*n* = 25) was the most prevalent. The predominant breed was Maltese (*n* = 17).

**Table 1:** Summary of signalment data

	Normal (*n* = 5)	Non-neoplastic hepatic disease (*n* = 30)	Hepatocellular carcinoma (*n* = 30)
Median age (range)	1	11 (6–15)	12.5 (6–17)
Sex (n)			
Female	0	2	2
Spayed female	0	12	12
Male	5	2	1
Castrated male	0	14	15
Breeds (n)	Beagle (5)	Maltese (9)	Maltese (8)
		Toy poodle (6)	Toy poodle (4)
		Shih-Tzu (3)	Shih-Tzu (4)
		Mixed (3)	Mixed (4)
		Yorkshire terrier (3)	Schnauzer (2)
		Pomeranian (1)	Pomeranian (2)
		Alaskan malamute (1)	Spitz (1)
		Labrador Retriever (1)	Cocker spaniel (1)
		Bichon Frise (1)	Scottish terrier (1)
		Spitz (1)	Yorkshire terrier (1)
		Dachshund (1)	French bulldog (1)
			Beagle (1)
Histology (n)	-	Inflammation (17)	-
	-	Vacuolar hepatopathy (11)	-
	-	Other (2)	-
Morphological subtype (n)	-	-	Massive (25)
	-	-	Nodular (4)
	-	-	Diffuse (1)

### MCL-1 expression in normal liver and HCC tissues

#### Western blotting

**Fig. 1:**
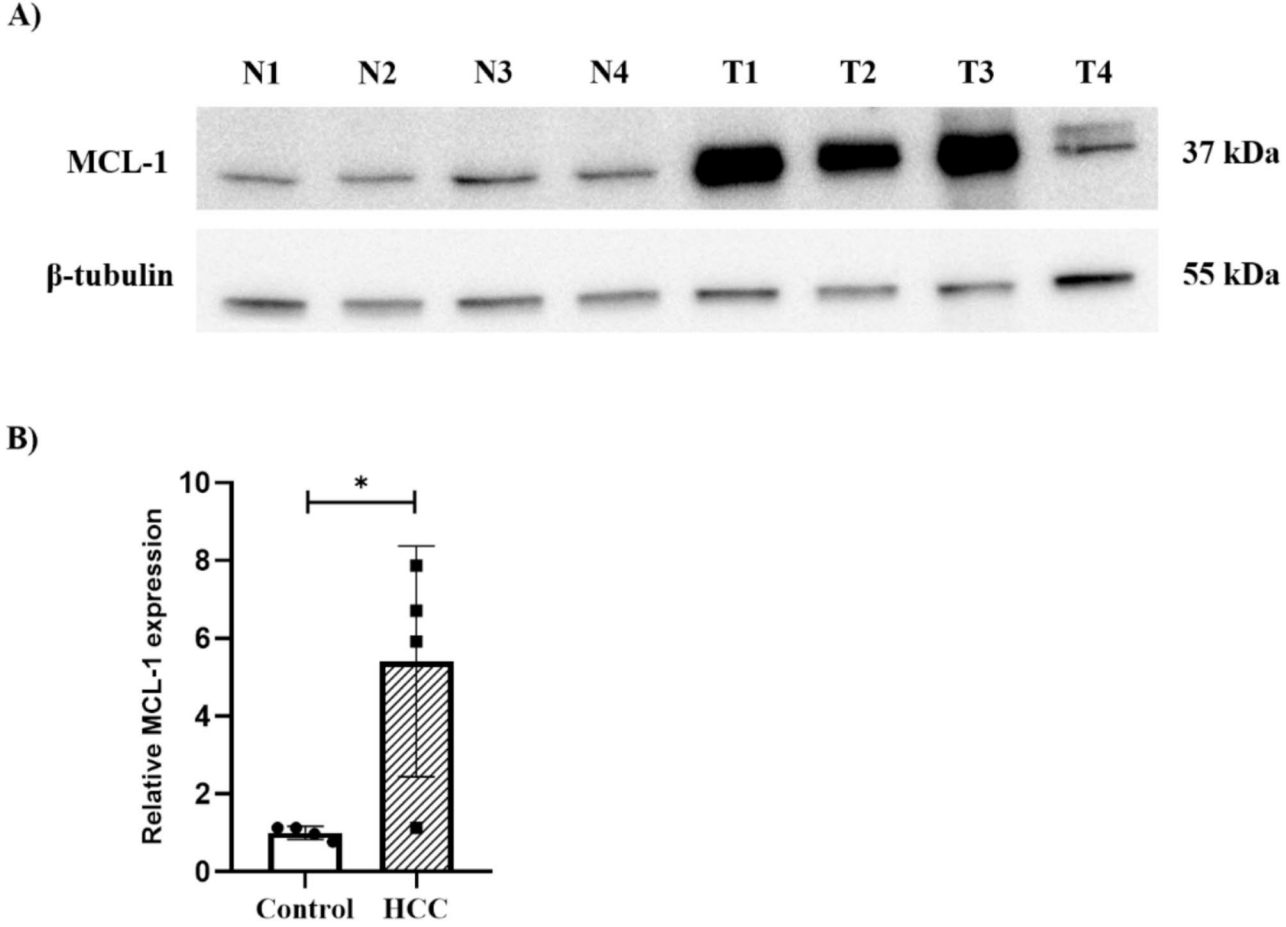
Comparison of MCL-1 expression in four normal and four HCC tissues. (**A**) Western blot analysis of MCL-1 protein (approximately 37 kDa) in normal and HCC tissues. β-tubulin (55 kDa) was used as the loading control. (**B**) Quantification of MCL-1 proteins was standardized relative to the control group. The mean ± standard error of quantitative data obtained from four tissues per group. (* *P* < 0.05). Full-length blots are presented in Supplementary Fig. 1. Abbreviations: N, normal; T, tumor; HCC, hepatocellular carcinoma; MCL-1, myeloid Cell Leukemia-1

MCL-1 expression (approximately 37 kDa) was confirmed in normal liver and HCC tissues, indicating the cross-reactivity of the antibody with canine liver tissue (Fig. 1A). β-Tubulin served as the loading control. For each sample, the band intensity of MCL-1 was quantified by densitometry and normalized to the corresponding β-tubulin band. The resulting MCL-1/β-tubulin ratios were averaged to establish a reference value, and relative fold changes were calculated by dividing each ratio by this baseline. This quantitative approach demonstrated a consistent and statistically significant upregulation of MCL-1 in HCC tissues compared to normal controls (*P* = 0.029) (Fig. 1B).

#### Immunohistochemistry (IHC)

**Fig. 2:**
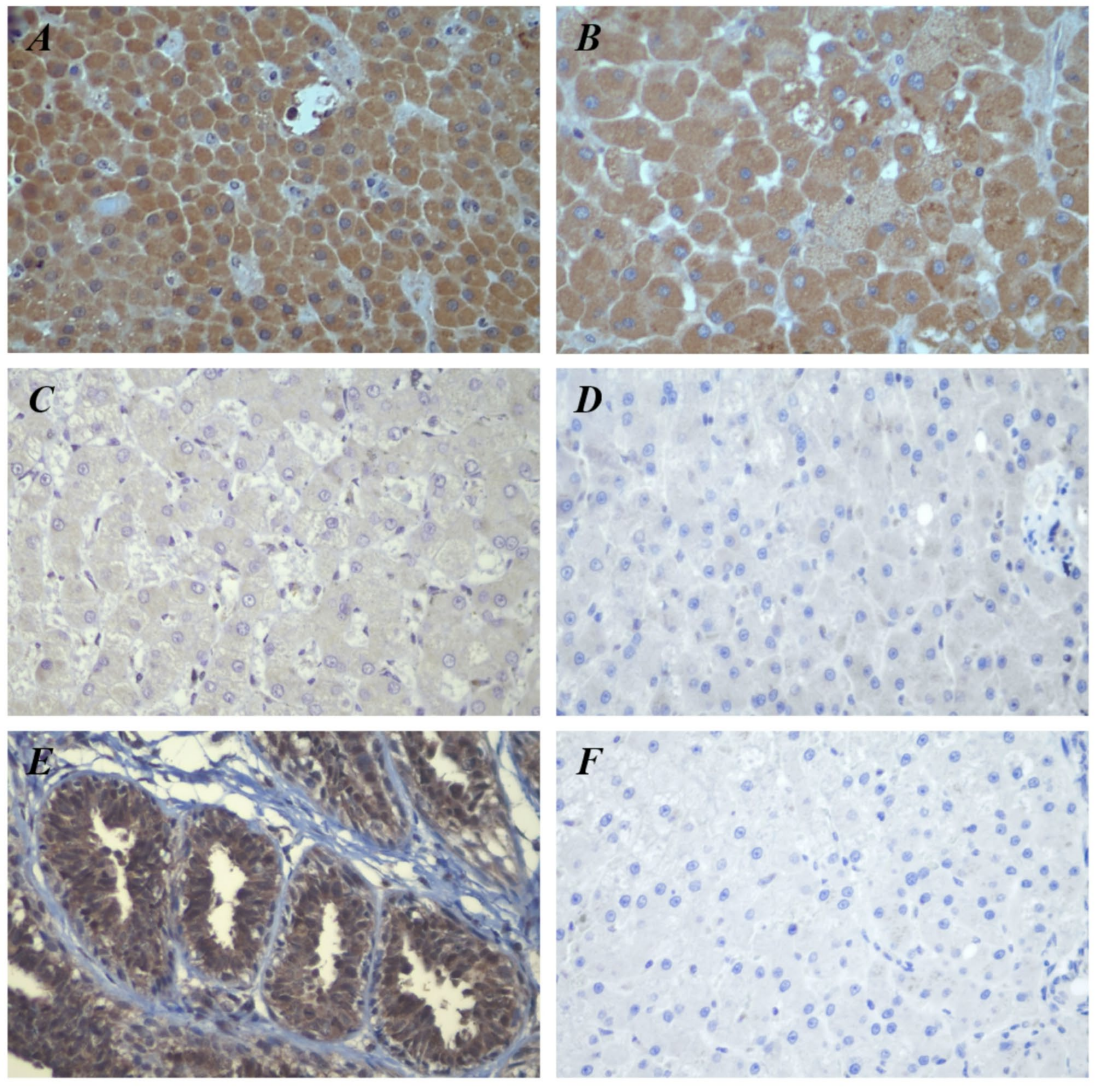
MCL-1 immunoreactivity in canine representative liver tissues. (**A**, **B**) HCC tissues demonstrating high MCL-1 immunostaining. (**C**) Non-neoplastic hepatic disease tissue demonstrating low MCL-1 immunostaining. (**D**) Normal canine liver tissue. (**E**) Positive control; MCL-1 expression in canine MGT tissue. (**F**) Negative control; no specific immunostaining was detected in normal liver tissue. (**A**-**F**: 400X magnification). Abbreviations: MCL-1, myeloid Cell Leukemia-1; HCC, hepatocellular carcinoma; MGT, mammary gland tumor

MCL-1 immunostaining was primarily detected in hepatocyte cytoplasm. As illustrated in Fig. 2A, B, MCL-1 was prominently expressed in HCC tissues. All 10 normal canine gland tissues exhibited low MCL-1 immunostaining (Fig. 2C). Immunostaining was more significant in non-neoplastic hepatic tissues than in normal tissues; however, the staining intensity was significantly lower than that observed in the HCC tissues. (Fig. 2A-D). High MCL-1 expression was noted in a remarkably high percentage of HCC tissues, and the proportion of high MCL-1 expression markedly increased as carcinogenicity progressed (*P* < 0.001) (Table 2).

**Table 2:** MCL-1 expression in normal livers, non-neoplastic hepatic diseases, and HCC

	Normal liver tissue *n* = 10 *n* (%)	Non-neoplastic hepatic diseases *n* = 30 *n* (%)	Hepatocellular carcinoma *n* = 30 *n* (%)	*P*-value
**MCL-1 expression**				
Low	10 (100)	23 (77)	9 (30)	*P* < 0.001*
High	0 (0)	7 (23)	21 (70)	

*Linear association test indicating a significant difference between tissue carcinogenicity and the expression level of MCL-1 (*P* < 0.05). Low vs. high was divided by the cut-off value obtained through the ROC curve

In the positive control, immunoreactivity was localized in the epithelial cells of the canine MGT (Fig. 2E) [22]. In contrast, negative controls using normal liver tissue showed no significant immunostaining (Fig. 2F).

#### Correlation of clinicopathological parameters and MCL-1 expression

Associations between MCL-1 expression and clinicopathological factors of HCC tissues are outlined in Table 3. The median age of dogs with high MCL-1 was 13 years, which is comparable to that of dogs with low MCL-1, whose median age was 11 years. No significant age-related differences were observed in MCL-1 expression levels (*P* = 0.898). In the HCC group, enhanced MCL-1 expression showed a positive correlation with tumor size > 5 cm (*P* = 0.046) and tumor metastasis (*P* = 0.034), which were documented as a poor prognosis [3, 25]. However, high MCL-1 expression did not demonstrate significant associations with well-established prognostic factors, including tumor location and liver enzyme level (ALT and AST) [3].

**Table 3:** Association of clinicopathological parameters and MCL-1 expression

	Number of tissues	MCL-1 expression		*P*-value
		Low	High	
**Hepatocellular carcinoma**				
Median age (range)	30	11 (9–13)	13 (6–17)	*P* = 0.197
Subtypes				*P* = 0.276
Massive	25	9	16	
Nodular	4	0	4	
Diffuse	1	0	1	
Tumor location				*P* = 0.146
Left	16	7	9	
Central	9	2	7	
Right	5	0	5	
Size of tumor				*P* = 0.046^*^
≤ 5 cm	9	5	4	
> 5 cm	21	4	17	
Blood analysis				*P* = 0.109
ALT				
Normal	5	3	2	
High	25	6	19	
AST				*P* = 0.936
Normal	17	5	12	
High	13	4	9	
ALP				*P* = 0.120
Normal	1	1	0	
High	29	8	21	
GGT				*P* = 0.690
Normal	15	5	10	
High	15	4	11	
Metastasis				*P* = 0.034^*^
Yes	12	1	11	
No	18	8	10	
Hepatic disease				
Vacuolar hepatopathy	11	10	1	*P* = 0.200
Inflammation	17	11	6	
Others	2	2	0	

*Chi-square test indicating significant relevance of high MCL-1 immunostaining and clinicopathological parameters (*P* < 0.05)

#### Survival curve

**Fig. 3:**
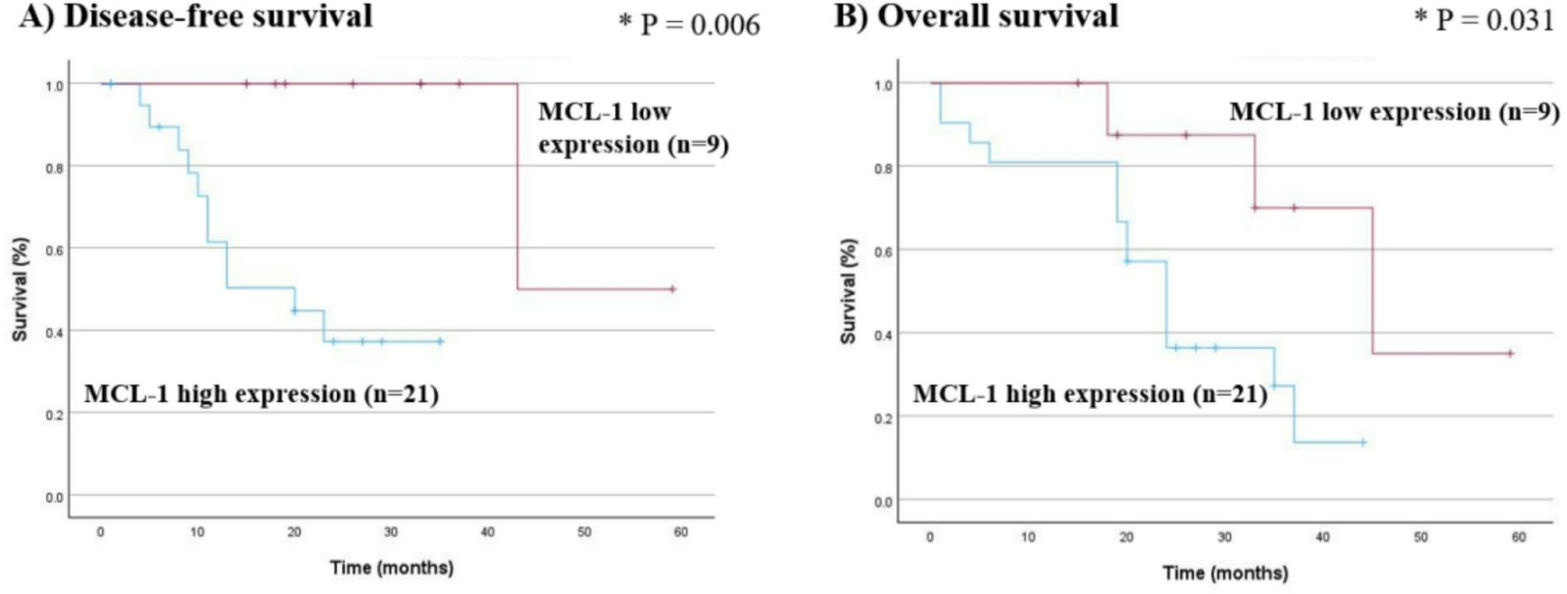
Kaplan–Meier curves for (**A**) disease-free survival and (**B**) overall survival in 30 HCC dogs based on MCL-1 immunostaining expression. The high MCL-1 expression group showed worse outcomes than the low MCL-1 expression group (*P* = 0.006 for DFS; *P* = 0.031 for OS). Abbreviations: MCL-1, myeloid Cell Leukemia-1; HCC, hepatocellular carcinoma

In this study of 30 patients with HCC, 18 died because of malignancy by the end of the study. The patients were divided into two groups—9 with low and 21 with high MCL-1 expression. Subsequently, Kaplan–Meier survival analysis with log-rank test was performed to evaluate the association between MCL-1 expression level and both OS and DFS. In the DFS analysis, eight dogs (89%) with low MCL-1 expression and 10 dogs (48%) with high MCL-1 expression were censored. In the OS analysis, six dogs (67%) with low MCL-1 expression and six dogs (29%) with high MCL-1 expression were censored. The survival curves demonstrated that overexpressed MCL-1 had significantly shorter median DFS (*P* = 0.006) and OS (*P* = 0.031) (Fig. 3). The median DFS and OS in the group with high MCL-1 were 20 and 24 months, respectively. To further determine whether MCL-1 expression independently influenced prognosis, a multivariate Cox proportional hazards regression analysis was conducted. High MCL-1 expression was identified as a significant independent factor of poor overall survival (HR = 5.853, 95% CI: 1.068–32.048, *P* = 0.042) (Table 4).

**Table 4:** Association of MCL-1 expression, tumor size, and metastasis with overall survival in HCC dogs: multivariate Cox proportional hazards regression

Variable	Hazard Ratio (HR)	95% Confidence Interval	*P*-value
MCl-1 (High vs. Low)	5.853	1.069–32.051	*P* = 0.042^*^
Tumor size (> 5 cm vs. ≤ 5 cm)	0.457	0.143–1.458	*P* = 0.186
Metastasis (Yes vs. No)	1.104	0.402–3.029	*P* = 0.848

Note: Low MCL-1 expression, tumor size ≤ 5 cm, and no metastasis were used as reference categories in the analysis

## Discussion

This study aimed to demonstrate that MCL-1 is overexpressed in canine HCC compared to normal and non-neoplastic hepatic disease tissues and investigate the association between enhanced MCL-1 expression and poor prognosis in HCC dogs. MCL-1 exists as multiple isoforms produced through alternative splicing, including MCL-1 L, MCL-1ES, and MCL-1 S [26, 27]. MCL-1 L, a significant anti-apoptotic isoform, is composed of 350 amino acids and includes all four BH domains, including BH1 and BH2, which are required to form the BH3-binding pocket. This structural configuration enables MCL-1 L to inhibit pro-apoptotic proteins, thereby facilitating cell survival [26, 28]. In this study, western blot analysis detected an immunoreactive band at approximately 37 kDa in normal canine liver tissues (Fig. 1A), consistent with the calculated molecular weight of 37.2 kDa for MCL-1 L [26, 27]. This finding supports the presence of the anti-apoptotic MCL-1 L isoform in the liver tissue, consistent with its role in regulating the apoptosis pathway.

Immunohistochemical studies have demonstrated that MCL-1 is observed in multiple normal human tissues, including hepatocytes [29] and in normal canine liver cells [30]. Although the precise MCL-1 function in liver tissue remains unclear, it is recognized as a significant factor in maintaining hepatocyte homeostasis and protecting hepatocytes from apoptotic pathways [11]. MCL-1 immunostaining was localized in the cytoplasm of canine liver tissue, which is consistent with a previous study demonstrating a granular pattern associated with intracellular organelles [19]. This cytoplasmic distribution aligns with its role in the mitochondrial membrane, where it inhibits MOMP to prevent apoptosis by interacting with apoptotic proteins (BAX and BAK) [8].

Unregulated apoptosis is a significant factor in cancer progression [14]. Cancer cells often exploit MCL-1 upregulation to evade cell death by neutralizing pro-apoptotic proteins to facilitate mitochondrial stabilization and enhance cell survival [26]. Our study demonstrated that MCL-1 intensity is significantly stronger in canine HCC tissues, indicating an association between MCL-1 upregulation and HCC progression [10]. Similarly, the immunostaining scores of MCL-1 were substantially higher in canine HCC tissues than those in normal and non-neoplastic hepatic disease tissues. These results align with previous reports in human HCC, where MCL-1 expression was more prominent in HCC tissues than in non-tumorous liver tissues [17–19]. Peyron et al. [31] reported that in dogs, alterations in mitochondrial ultrastructure, metabolism, and keratin filaments in vacuolar hepatopathy-affected livers. Comparable structural and metabolic liver alterations have been observed in humans with nonalcoholic steatohepatitis, a well-known risk factor for HCC [32]. Vacuolar hepatopathy in Scottish terriers can be associated with HCC development [33]. This indicates that vacuolar hepatopathy-related conditions in dogs may increase the risk of HCC development. Additionally, although rare, the advancement of chronic hepatitis in dogs to later stages may be associated with HCC onset [34]. In this study, tissue immunostaining intensity demonstrated an increasing pattern, with the lowest expression levels observed in normal tissues, followed by non-neoplastic disease tissues, and the highest levels in HCC. Therefore, progressive enhancement in MCL-1 expression may be linked to tissue carcinogenesis. In human HCC, MCL-1 expression is regulated by the phosphoinositide 3-kinase (PI3K) pathway [17, 19]. PI3K expression is significantly enhanced in HCC tumor tissues and is associated with enhanced tumor proliferation and reduced apoptosis. Additionally, HCC patients with high PI3K expression are associated with a worse prognosis [35]. PI3K inhibition results in a rapid reduction in MCL-1 levels [19], indicating a positive correlation between enhanced PI3K expression and MCL-1 overexpression in HCC. A comparable pathway may be involved in the progression of canine HCC; however, additional studies are required to elucidate the precise mechanism of MCL-1.

Here, we demonstrated that high MCL-1 expression was correlated with metastasis and tumor size, which are prognostic markers of canine HCC. Tumor diameter > 5 cm and the presence of metastasis are associated with significantly worse outcomes [3, 25]. A high MCL-1 expression in canine MGT has been associated with larger tumor size and metastasis [22]. This relationship can be attributed to the function of MCL-1 overactivation in inhibiting the apoptosis process. Additionally, we demonstrated that elevated MCL-1 immunostaining is associated with worse survival rates. Notably, multivariate Cox regression analysis confirmed that high MCL-1 expression was a significant independent prognostic factor, even after adjusting for tumor size and metastasis. This finding underscores the prognostic relevance of MCL-1 beyond its correlation with other clinical variables. MCL-1 overexpression in humans is correlated to poor prognosis in numerous cancers, such as gastric cancer, lung cancer, and hematological malignancies [13–16]. Based on these findings, our study proposes that MCL-1 protein can be used as a novel prognostic marker in canine HCC.

Sorafenib is an FDA-approved compound used to treat advanced HCC in humans [36]. Although it is best known as a multi-kinase inhibitor, studies have demonstrated that it inhibits protein translation, resulting in the downregulation of MCL-1 expression in HCC cell lines [37, 38]. Similarly, Sorafenib has been used to treat unresectable HCC in canine patients [6]. Our study demonstrated that canine HCC exhibits enhanced MCL-1 expression. Because Sorafenib can effectively reduce MCL-1 expression, our findings may provide additional evidence that Sorafenib can be a potential treatment option for advanced canine HCC. However, further research is required to confirm its role in regulating MCL-1 expression in canine HCC. Additionally, therapeutic approaches that target MCL-1 are being explored across various cancers, including HCC [10, 20]. Specifically, combining MCL-1 inhibitors with other chemotherapeutic regimens can potentially overcome intrinsic apoptosis resistance, enabling more precise, biomarker-driven treatment approaches [10, 20]. Consistent with human studies, MCL-1 may be a valuable therapeutic target in canine HCC. The present study may serve as an initial step in validating the MCL-1-associated mechanisms and potential therapies.

This study is limited by the relatively small number of canine HCC samples. In particular, only four representative samples of normal and HCC tissues were included in the Western blot analysis due to the limited availability of fresh-frozen tissues with sufficient protein integrity. These samples were carefully selected to reflect clinical heterogeneity, including tumor size and the presence or absence of metastasis, to ensure representative coverage despite the small number. The limited sample size also prevented a robust evaluation of significant associations between MCL-1 expression and documented prognostic factors, including age, liver enzyme levels (ALT and AST), subtypes, and tumor location [3, 39]. Future studies should include a larger number of high-quality, fresh-frozen samples for Western blot analysis to improve the reliability of quantitative protein expression data and to further validate the role of MCL-1 in canine HCC. Obtaining samples for nodular or diffuse subtypes has also been challenging because the main operable subtype of HCC is the massive form. Nevertheless, a recent study demonstrated that there was no significant difference in prognosis based on morphological subtype [40]. Incorporating a broader spectrum of morphological subtypes in future studies will be crucial for enhancing the external validity of findings and facilitating a more comprehensive and multi-perspective analysis.

## Conclusions

We confirmed the presence of MCL-1 protein in canine liver tissue using IHC and western blot analysis. HCC tissues exhibited significantly higher MCL-1 expression than normal canine liver tissues and tissues from non-neoplastic hepatic disease. Higher MCL-1 expression was significantly associated with metastasis and tumor size. Finally, upregulated MCL-1 expression had poor survival outcomes. These findings suggest new possibilities for MCL-1 to be used as a useful diagnostic and prognostic marker. However, further research is required to clarify the role of MCL-1 in the progression of canine HCC.

## Electronic supplementary material

Below is the link to the electronic supplementary material.

Supplementary Material 1

## Data Availability

The data used in this study are available from the corresponding author upon reasonable request.
