# Supplementary material for: Overexpression of MCL-1 in canine hepatocellular carcinoma and its efficacy as a prognostic marker
Source: BMC Vet Res. 2025 May 16;21:349. doi: 10.1186/s12917-025-04798-6 (PMC12082996; doi:10.1186/s12917-025-04798-6)
Supplement: Supplementary file 1 — Supplementary Material 1 [file 12917_2025_4798_MOESM1_ESM.pdf]

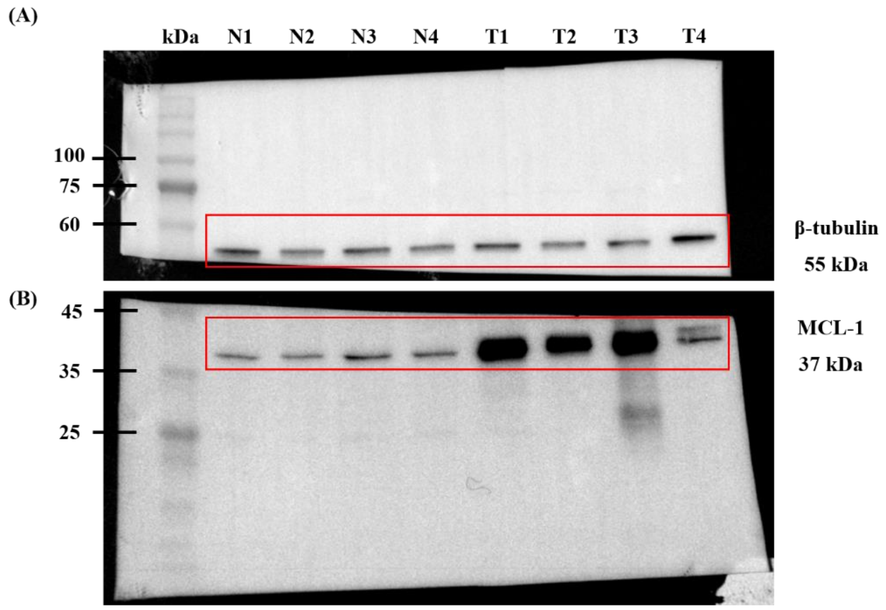

Supplementary Figure 1. (A) Full length blot of β -tubulin antibody. (B) Full length blot of MCL-1 antibody. Red boxes indicate the cropped blots shown in Figure 1. of this study. Protein marker (PM2700, SMOBIO, ExcelBand^{TM} 3-color Broad Range Protein Marker). Abbreviation: N, normal liver tissue, T, tumor tissue (hepatocellular carcinoma), MCL-1, myeloid cell leukemia-1.
